# A core phylogeny of Dictyostelia inferred from genomes representative of the eight major and minor taxonomic divisions of the group

**DOI:** 10.1186/s12862-016-0825-7

**Published:** 2016-11-17

**Authors:** Reema Singh, Christina Schilde, Pauline Schaap

**Affiliations:** School of Life Sciences, University of Dundee, MSI complex, Dow Street, Dundee, DD15EH UK

**Keywords:** Multi-locus phylogeny, Dictyostelia, Phylogenomics, Evolution of soma, Evolution of multicellularity, Taxonomy

## Abstract

**Background:**

Dictyostelia are a well-studied group of organisms with colonial multicellularity, which are members of the mostly unicellular Amoebozoa. A phylogeny based on SSU rDNA data subdivided all Dictyostelia into four major groups, but left the position of the root and of six group-intermediate taxa unresolved. Recent phylogenies inferred from 30 or 213 proteins from sequenced genomes, positioned the root between two branches, each containing two major groups, but lacked data to position the group-intermediate taxa. Since the positions of these early diverging taxa are crucial for understanding the evolution of phenotypic complexity in Dictyostelia, we sequenced six representative genomes of early diverging taxa.

**Results:**

We retrieved orthologs of 47 housekeeping proteins with an average size of 890 amino acids from six newly sequenced and eight published genomes of Dictyostelia and unicellular Amoebozoa and inferred phylogenies from single and concatenated protein sequence alignments. Concatenated alignments of all 47 proteins, and four out of five subsets of nine concatenated proteins all produced the same consensus phylogeny with 100% statistical support. Trees inferred from just two out of the 47 proteins, individually reproduced the consensus phylogeny, highlighting that single gene phylogenies will rarely reflect correct species relationships. However, sets of two or three concatenated proteins again reproduced the consensus phylogeny, indicating that a small selection of genes suffices for low cost classification of as yet unincorporated or newly discovered dictyostelid and amoebozoan taxa by gene amplification.

**Conclusions:**

The multi-locus consensus phylogeny shows that groups 1 and 2 are sister clades in branch I, with the group-intermediate taxon *D. polycarpum* positioned as outgroup to group 2. Branch II consists of groups 3 and 4, with the group-intermediate taxon *Polysphondylium violaceum* positioned as sister to group 4, and the group-intermediate taxon *Dictyostelium polycephalum* branching at the base of that whole clade. Given the data, the approximately unbiased test rejects all alternative topologies favoured by SSU rDNA and individual proteins with high statistical support. The test also rejects monophyletic origins for the genera *Acytostelium*, *Polysphondylium* and *Dictyostelium.* The current position of *Acytostelium ellipticum* in the consensus phylogeny indicates that somatic cells were lost twice in Dictyostelia.

**Electronic supplementary material:**

The online version of this article (doi:10.1186/s12862-016-0825-7) contains supplementary material, which is available to authorized users.

## Background

The invention of multicellularity is considered to be one of the major evolutionary transitions [1], but is by no means a rare event. Multicellular forms appeared at least 11 times independently in most eukaryote divisions and in prokaryotes [2–4]. The better known lineages, such as animals, fungi, green plants and red- and brown macroalgae evolved a form of multicellularity where cells stay together after cell division. However, the other forms mostly start off with foraging single cells that achieve multicellularity by aggregation, usually when starved or otherwise stressed. The aggregate then transforms into a fruiting structure with an elevated mass of dormant spores or cysts. Thus far, the social amoeba *Dictyostelium discoideum* shows the most complex form of aggregative multicellularity with an intermediate migrating “slug” stage and cells specializing into spores and three somatic cell types that form a stalk and structures to support the stalk and spore mass. *D. discoideum* is also a popular model organism for studying cellular processes such as cell migration [5], cytokinesis, vesicle trafficking, host-pathogen interactions [6], cell differentiation and morphogenesis [7], as well as evolution of sociality and prey–predator relationships [8, 9]. We aim to identify the molecular changes that caused the transition to multicellularity and the acquisition of phenotypic complexity during dictyostelid evolution. In collaboration with Baldauf and coworkers, we initiated this research by constructing the first molecular phylogeny of the then 99 known Dictyostelia using SSUrDNA and α-tubulin sequence data [10]. Phylogenies derived from either gene subdivided Dictyostelia into four major groupings, but did not agree on the position of the root and of several group-intermediate taxa. The position of taxa at terminal branches was also poorly resolved. The latter was improved by sequencing of the less conserved internal transcribed spacer (ITS) regions between the ribosomal RNAs [11]. Further expansion of the phylogeny with 47 new taxa indicated that the intermediate species may actually represent additional minor groups, which were named the violaceum, polycephalum and polycarpum complexes [12].

The availability of the phylogeny allowed us and others to select group-representative taxa for genome sequencing in addition to the *D. discoideum* genome, which was already available [13]. These taxa are *D. purpureum* [14], like *D. discoideum* in group 4. *D. lacteum* in group 3 [15], *Polyspondylium pallidum* in group 2, clade 2B [16], *Acytostelium subglobosum* in group 2 clade 2A [17] and *D. fasciculatum* in group 1 [16]. The sequenced genomes enabled phylogenetic inference from multiple concatenated protein sequences and these efforts, using respectively 32 [18] and 213 protein sequences [19], both repositioned the root from a location between groups 1 and 2 to a location that separated the four groups into two branches, with branch I containing groups 1 and 2 and branch II, groups 3 and 4. Since no genome sequences for group-intermediate species were available, their positions remained unresolved.

Mapping of phenotypic characters onto the phylogeny revealed that groups 1, 2 and 3 share many common features, such as small clustered or branched fruiting bodies with stalks being formed by dedifferentiation of prespore cells, use of glorin as attractant for aggregation and retention of encystation, the survival strategy of unicellular amoebas [18, 20]. Group 4 taxa generally form large, solitary and unbranched fruiting bodies, and set aside a proportion of specialized cells to form the stalk. Group 4 taxa furthermore use cAMP as attractant, have lost encystation and acquired a migrating slug stage, while many taxa in the group acquired one or two novel cell types. Appropiate understanding of the evolutionary history of these major innovations requires correct understanding of the relationship between groups 3 and 4 and the group-intermediate taxa. The SSU rDNA phylogeny subdivides group 2 into two clades, of which one (2A) uniformly lacks the cellular stalk. However, one taxon (*A. ellipticum*) that lacks stalk cells groups with the other clade (2B), suggesting that the cellular stalk was gained twice, which seems unlikely [10]. Because the intermediate species link the deepest nodes in the tree, their position is also crucial for understanding the earliest processes that triggered the transition from uni- to multicellularity in Dictyostelia.

Proper reconstruction of phenotypic evolution and its association with changes at the gene and genome level critically depends on a correct understanding of the positions of the group-intermediate taxa. We therefore sequenced the genomes of one taxon each of the violaceum, polycarpum and polycephalum complexes. Additionally, we sequenced the genome of *A. ellipticum*, with its problematic placement in clade 2B, and to better outline clade 2A, *A. leptosomum,* which is in the SSU rDNA phylogeny most distant from *A. subglobosum*, with an already sequenced genome. We also sequenced the genome of *D. deminutivum*, the earliest diverging species in group 1 with the most primitive multicellular features. We retrieved 47 orthologous proteins from all dictyostelid genomes and three sequenced genomes of unicellular Amoebozoa and inferred phylogenies from concatenated alignments of all proteins and various subsets by different methods. This yielded a very robust consensus phylogeny of Dictyostelia with well defined positions for the group-intermediate taxa.

## Results

### Genome sequencing of early diverging dictyostelid taxa

To improve the internal node structure of the dictyostelid phylogeny, the genomes of four group-intermediate species and two other early diverging *Dictyostelium* species were sequenced, using the Illumina platform. Despite careful washing and 4 h starvation to rid cells of their bacterial food source, the genome reads of two species, *P. violaceum* (*Pvio*) and *A. leptosomum* (*Alep*) consisted for respectively 78% and 39% of *E.coli* sequences (Additional file 1). The other genomes of *D. deminutivum* (*Ddem*), *D. polycephalum* (*Dcep*), *A. ellipticum* (*Aell*) and *D. polycarpum* (*Dcar*) contained from 0.5 to 19% *E.coli* reads. With all species being prepared for gDNA extraction in the same manner, it is not clear what caused this large variation in contamination with genomic DNA from their food source. Possibly, *Pvio,* strain P6 and *Alep* are “farmers”, that retain bacteria in their fruiting bodies for future cultivation [21].

We retrieved mostly complete genes for all of our selected test proteins from the *Ddem*, *Dcep*, *Aell* and *Dcar* genomes. However, BLAST searches of the *Pvio* genome returned many prokaryote hits, while for the *Alep* genome, the orthologous sequences were often incomplete. Fortunately, genome contig sequences for *Pvio* strain QSvi11 were made available in Genbank by BCM-HGSC (https://www.hgsc.bcm.edu/) under accession number AJWJ00000000, which proved to be of good quality. The retrieved *Alep* sequences still contributed to 45% of the concatenated aligned sequences, which was sufficient to place *Alep* at its expected position as sister to *A. subglobosum (Asub)*.

### Protein selection, alignment and phylogenetic inference

We selected 40 proteins from 14 primary metabolic pathways and 12 proteins with a range of roles in basic cell biology from the *D. discoideum* (*Ddis*) genome with a size >200 amino acids. This selection of proteins from a broad spectrum of metabolic and cellular roles

was intended to average out any effects of natural selection on gene polymorphisms in concatenated alignments. The setting of a minimum size to the selected proteins was meant to i. avoid underrepresentation of the phylogenetic signal from very small proteins and ii. to provide opportunities to select some proteins that individually reproduce the consensus phylogeny for a gene amplification approach for classification of a broad range of taxa.

**Fig. 1:**
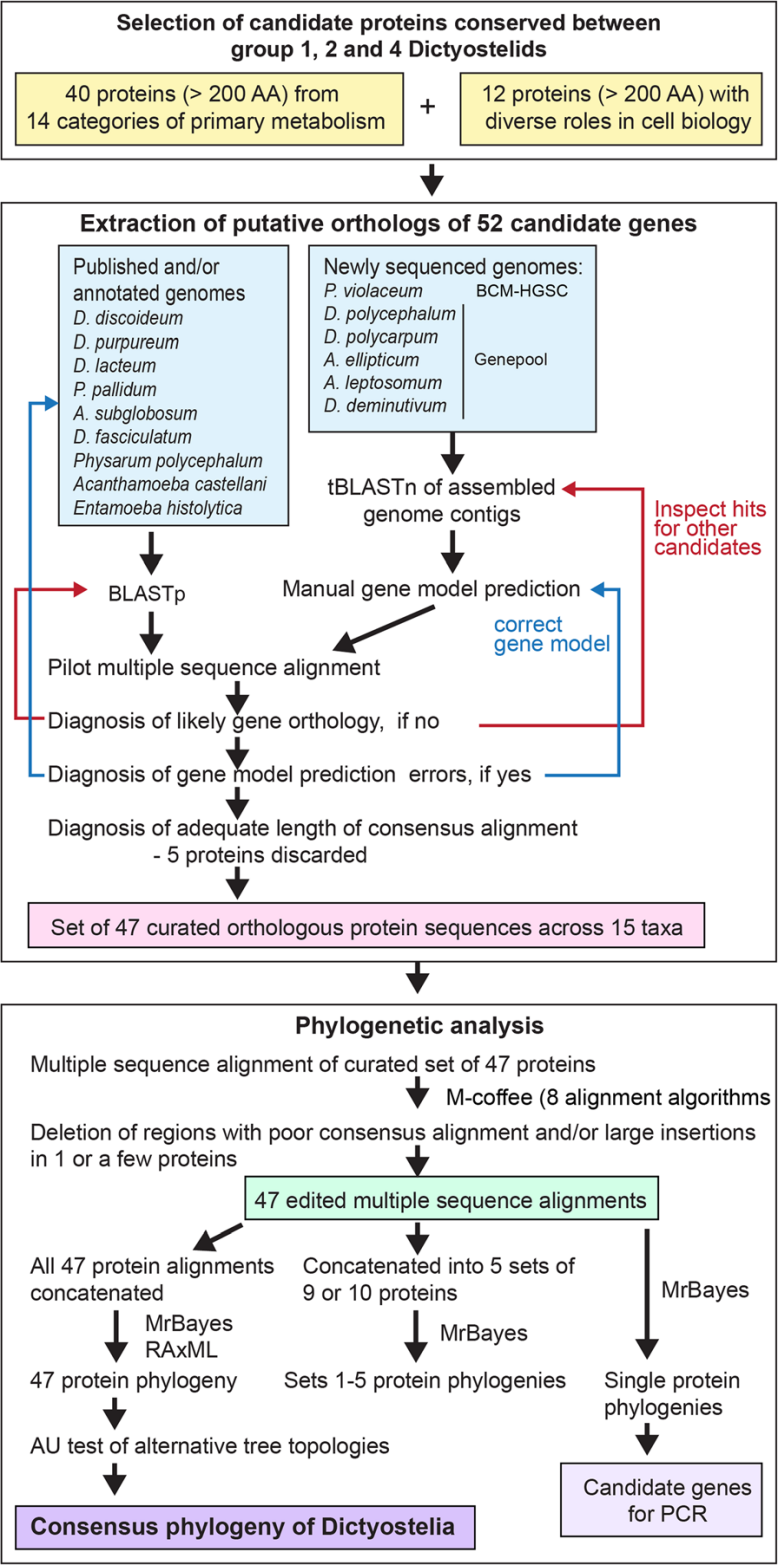
Bioinformatics pipeline. Chain of procedures for protein selection, cognate gene identification, gene model prediction and phylogenetic analysis

We used BLASTp to search for orthologs of the 52 proteins sequences in the annotated genomes of *D. purpureum (Dpur)*, *D. lacteum (Dlac)*, *P. pallidum (Ppal)*, *D. fasciculatum (Dfas)*, *Asub*, *Acanthamoeba castellani (Acas)*, *Physarum polycephalum* (*PhyP*) and *Entamoeba histolytica* (*Ehis*) and we used tBLASTn to find orthologous genes in the newly sequenced *Dcar*, *Dcep*, *Aell*, *Alep* and *Ddem* genomes and the unannotated *Pvio* QSvi11 genome. For the latter genomes, gene models were manually predicted, assisted by the models of the orthologous genes in the annotated genomes. See the analysis pipeline in Fig. 1.

The individual orthologous proteins were aligned and all sections that did not align unambiguously as well as large insertions in individual proteins were deleted. Five proteins which showed only small sections of consensus alignment were rejected from the set. The alignments of the remaining 47 proteins were concatenated fully, and into five sets of nine or ten proteins (see Additional file 2, sheet 3). All sets as well as alignments of the individual protein sequences were used for phylogenetic inference.

**Fig. 2:**
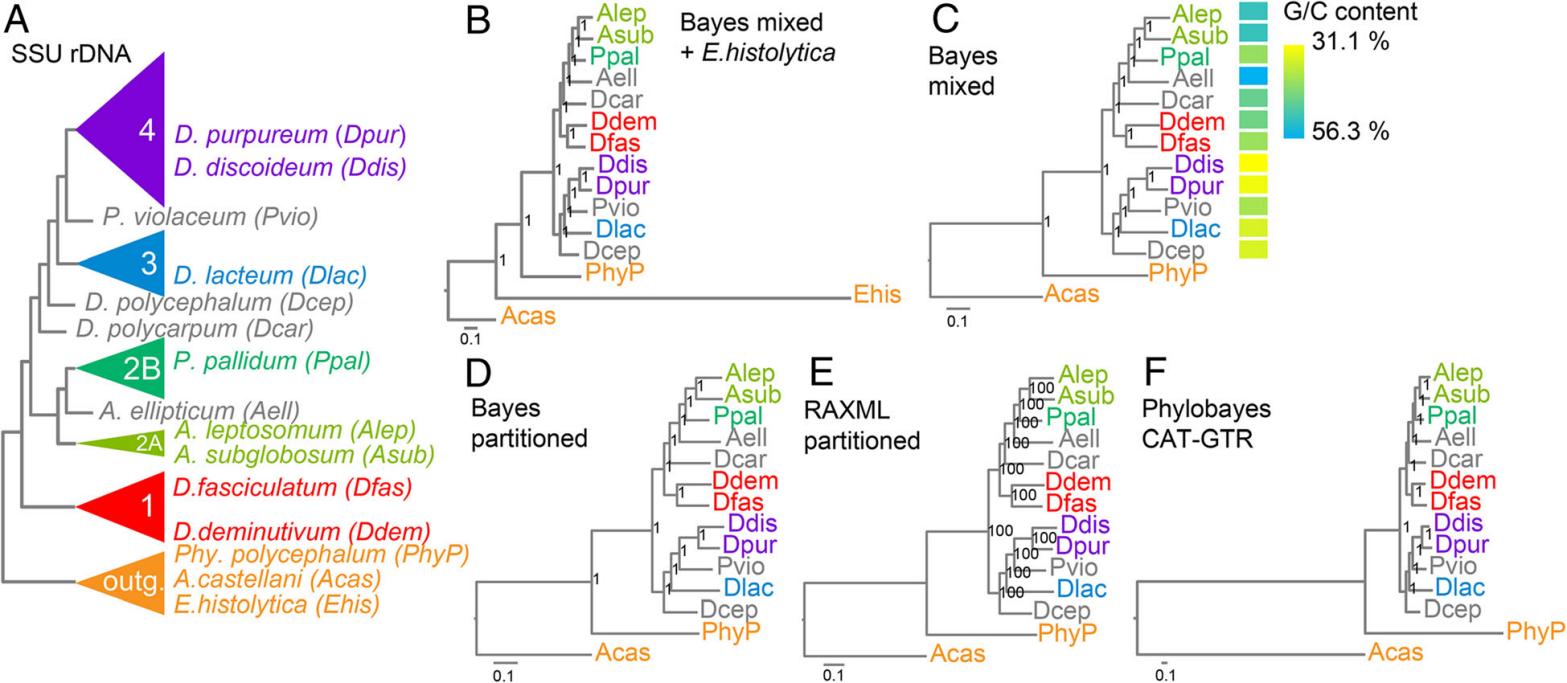
Phylogenetic inference from 47 concatenated proteins. **a** Location of selected species in the previously inferred SSU rDNA phylogeny [10]. **b**–**f** Phylogenetic trees inferred by Bayesian inference (**b**–**d**), RAxML (**e**) or Phylobayes (**f**) from an alignment of 47 concatenated orthologous proteins that were identified in the species shown in **a**. Bayesian analyses were run for 100,000 generations with either a mixed amino-acid model (**b**,**c**) for the entire alignment, or with a partitioned alignment in which each protein was run under its optimal amino-acid substitution model (**d**). All analyses converged within 6000 generations (SD of split frequencies = 0). The RaxML analysis was run with 100 bootstrap replicates on an alignment partitioned as in **d**. Phylobayes MPI [22] was run over two chains under the CAT-GTR model (**f**). Trees were rooted using *A. castellani* as outgroup. The average GC content of the genomic DNAs encoding the 47 proteins is plotted onto the phylogeny in panel **c**. Posterior probabilities or bootstrap support for the nodes are shown. Abbreviated and full species names are colour-coded to reflect the taxon group to which the species belong

Bayesian inference of all 47 concatenated proteins using a mixed amino acid substitution model yielded a different topology from the previous SSU rDNA tree (Fig. 2a,b). Firstly, as in the published multi-protein phylogenies [18, 19], the root is no longer positioned between groups 1 and 2, but separates two branches containing groups 1 and 2 and groups 3 and 4. Additionally, the group-intermediate species *Dcar* changed position from the base of the clade comprising groups 3 and 4 to the base of group 2, while *Aell* which was previously sister to clade 2B is now branching at the base of the clade composed of 2A and 2B. Due to the repositioned root, groups 1 and 2 are now separate sister groups, while group 4 remains in a clade with group 3. We included the obligate parasite *Ehis* in the initial phylogeny, but as also found previously [18, 19], most *Ehis* genes are either absent or very divergent from those in other Amoebozoa. Because this could lead to long-branch attraction errors, *Ehis* was omitted from further analysis. This had no effect on tree topology (Fig. 2c).

Instead of running a mixed model on the entire concatenated alignment, we also partitioned the alignment to combine proteins with the same optimal amino-acid substitution model into one partition and then ran each partition under its optimal model. This returned the same phylogeny as the analysis with the mixed model (Fig. 2d). The use of maximum likelihood based tree inference on a similarly partitioned alignment also returned the same phylogenetic tree (Fig. 2e) as did inference by Phylobayes under the CAT-GTR model (Fig. 2f). In the latter model both the individual amino-acid substitution propensities and substitution rates are inferred from the underlying alignment [22]. The nodes in all trees have Bayesian posterior probabilities of 1.0 or 100% bootstrap support. The Bayesian analyses all converged to SD of split frequencies = 0 within 6000 generations. All these parameters indicate that the trees derived from the concatenated alignment are extremely robust.

In the course of gene model prediction, we noted extreme differences in the G/C content of the *Dictyostelium* genes. Plotting of the averaged G/C content of the 47 test genes onto the phylogeny showed that the branch I genes are more G/C rich than those in branch II, with Acytostelids being particularly G/C-rich and the two group 4 taxa being very A/T- rich (Fig. 2c).

### Trees from protein subsets and individual proteins

**Fig. 3:**
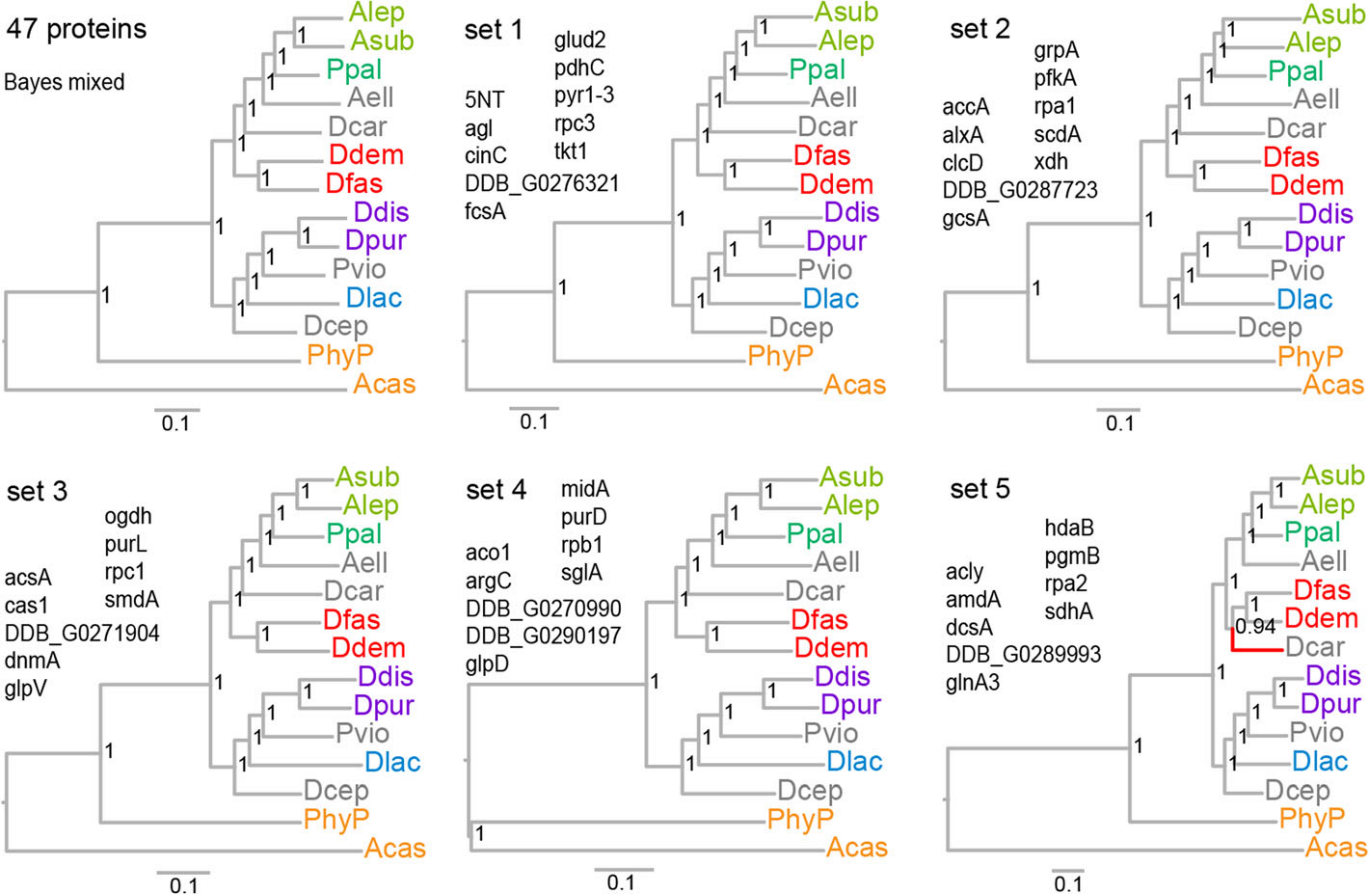
Phylogenetic inference from subsets. The 47 protein set was subdivided in sets of nine or ten proteins by joining the fifth rows of five staggered columns of protein identifiers. The protein alignments were concatenated per set, and each alignment was subjected to Bayesian inference with a mixed amino-acid model. Only set 5 yielded a tree topology that was different (red branch) from the 47 protein consensus topology (top left)

We next assessed to what extent smaller subsets of the concatenated protein sequences returned robust consensual phylogenies. To retain the functional diversity of proteins also in the subset, we subdivided 47 protein set in five sets of nine or ten proteins by joining the fifth proteins in the gene list (Additional file 2) five times in staggered fashion. Figure 3 shows that sets 1 to 4 yielded trees with identical topologies to the tree inferred from all 47 proteins, further referred to as “the consensus topology”, and also with probabilities of 1.0 for all nodes. Only the tree derived from set 5 showed a single divergent node with a probability of 0.94.

Trees were also inferred by Bayesian inference for all individual protein alignments (Additional file 3). Only two out of the 47 trees displayed the consensus topology (Additional file 2, sheet 4), while 12, 18, 14 and 1 tree(s) show 1, 2, 3 or 4 alternative node configurations respectively. The most common alternative topology was *Dcar* as outgroup to group 1 instead of group 2 (13 proteins) and permutations in the relative positions of *Pvio* and *Dlac* (nine proteins) (Additional file 2, sheet 5). Proteins that yielded trees with only one non-consensual node yielded consensus trees when concatenated with a second protein that yielded trees with a different error in four tested cases, while one protein, aco1, required two other proteins to produce a consensus tree (Additional file 4).

**Fig. 4:**
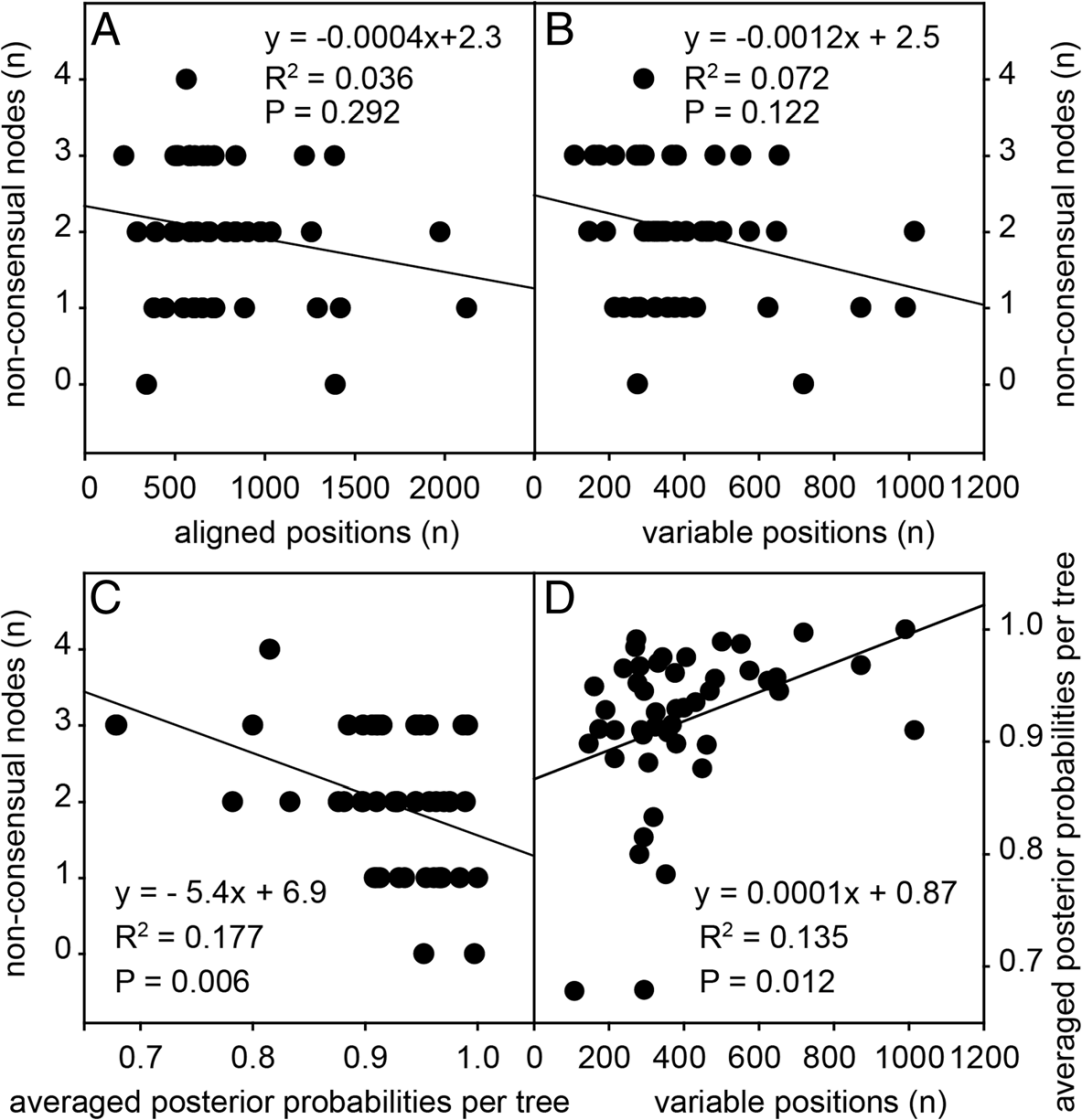
Correlations between protein alignment statistics and tree features. For trees inferred from alignments of orthologs for each of the individual 47 test proteins (Additional file 3), the number of non-consensual nodes in each tree was plotted against either the number of aligned (**a**) or variable (**b**) positions per alignment, or against the averaged posterior probabilities of all nodes in the tree (**c**). Averaged posterior probabilities were also plotted against the number of variable positions per alignment (**d**). Regression lines for all plots are shown with their equations and coefficients of determination. Correlations between the variables were determined by Spearman rank order and *P*-values are shown. All variables are listed in Additional File 2, sheet 4

To gain insight into parameters that render some proteins more suitable than others for taxonomy, we plotted the number of non-consensual nodes per tree against the number of aligned positions or variable positions, and against the averaged node posterior probabilities. There was only a very weak negative correlation between the number of aligned positions and the number of non-consensual nodes, which became slightly stronger, but still statistically insignificant, when only the variable positions in the alignment were considered (Fig. 4a,b). Only 7% of the variance in the number of non-consensual nodes is explained by the variance in the number of variable positions in the alignment, which implies that the remaining 93% is dependent on some unknown intrinsic property of the protein sequence itself.

There was a significant negative correlation between the number of non-consensual nodes and the averaged node probabilities (Fig. 4c), as well as a significant positive correlation between the number of variable positions and the averaged node probabilities per tree (Fig. 4d). Altogether this indicates that while longer alignments with more variable positions produce better supported trees, these trees are not necessarily more accurate in assessing relationships between species. We could not detect significant differences between the number of non-consensual nodes per protein tree and participation of the protein in a specific metabolic pathway (Additional file 2, sheet 6), but this could be due to the small number of proteins per pathway.

### Tests for alternative hypotheses

**Fig. 5:**
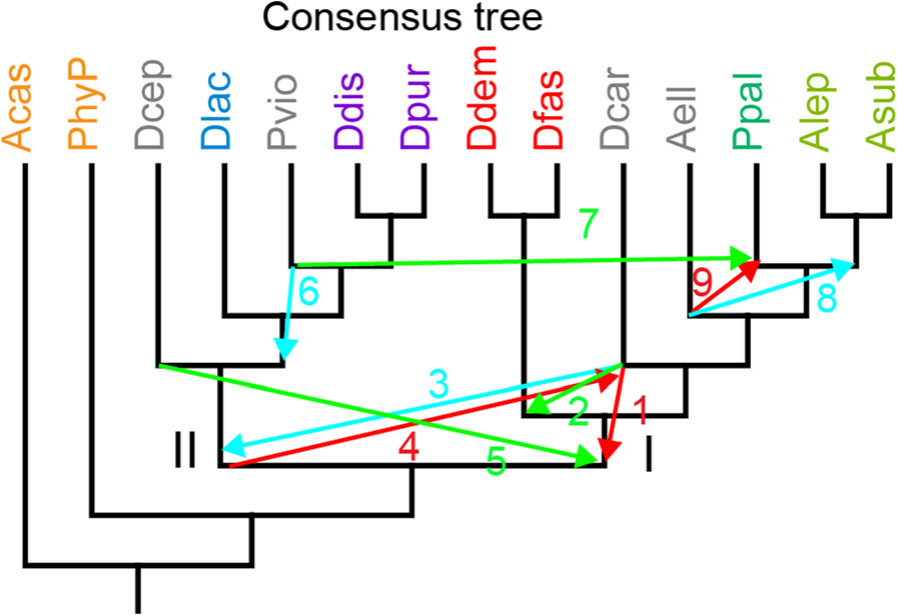
Alternative tree topologies. Schematic of repositioning of tree branches in the 47 protein consensus tree to yield the most commonly encountered alternative topologies found in either the earlier SSU rDNA tree of Dictyostelia [10], or the trees inferred from single proteins (Additional file 3). All alternative trees are shown in Additional file 5

While the full set of 47 proteins and four out of five sets of nine or ten proteins produced identical consensus core phylogenies for the Dictyostelia with 100% statistical support, most trees derived from individual proteins and the tree derived from the 5^th^ set of nine proteins produced a range of alternative topologies, as did the original SSU rDNA tree. We used the Approximately Unbiased (AU) test, implemented in the CONSEL software [23] to compare the confidence levels of the consensus tree topology and the most commonly encountered alternative topologies (Fig. 5, Additional file 5). Table 1 shows that alternative topologies such as the root position of the earlier SSU tree [10], monophyletic origins for Acytostelids and Polysphondylids and alternative positions for the group-intermediate species Dcep and Pvio are all strongly rejected. Only alternative placement of Dcar as outgroup to group 1 or branch I, instead of group 2 comes with a probability of 1:10,000 somewhat closer to the consensus topology with a probability of 1.0. We are therefore confident that the consensus is correct.

**Table 1:** Alternative topology tests

	Alternative topologies	*P*-value
Consensus	Unconstrained 47 protein phylogeny	1.000
Topology 1	*D. polycarpum* is outgroup to branch I	1e-04
Topology 2	*D. polycarpum* is outgroup to group 1	1e-04
Topology 3	*D. polycarpum* is outgroup to branch II	6e-74
Topology 4	Root between group 1 and group 2, as in SSU rDNA tree	5e-74
Topology 5	*D. polycephalum* is outgroup to branch I	3e-68
Topology 6	*P. violaceum* is outgroup to group 3	2e-43
Topology 7	*P. violaceum* groups with *P. pallidum*	4e-29
Topology 8	*A. ellipticum* groups with other Acytostelids	5e-47
Topology 9	*A. ellipticum* groups with Polysphondylids, as in SSU rDNA tree	5e-40

The probabilities (*p*-value) of the alternative tree topologies listed in Fig. 5 were investigated using the approximately unbiased (AU) test in CONSEL v0.20 [25]. Trees with constrained nodes were generated using RaxML and log likelihood values for the consensus tree and alternative trees were used as input for the AU test

## Discussion

To understand the directionality of evolutionary processes, a correct understanding of the genetic relationships between species is essential. For Dictyostelia, the relationships between the four major groups were well defined previously from multi-locus phylogenies [18, 19], but the positions of early diverging species that occupy intermediate outgroup positions to the major groups were poorly resolved. Their position is however of crucial importance to identify the molecular changes that triggered the phenotypic diversification of the major groups. To reliably position the intermediate groups, we sequenced the genomes of six early diverging species, and used the information to identify around 50 well-conserved proteins for phylogenetic inference.

The final set of 47 proteins has only one or seven proteins in common with sets of 32 or 213 proteins, respectively, that were used previously to root the dictyostelid phylogeny [18, 19]. Nevertheless, phylogenies inferred from all three sets position the root between branch I, containing groups 1 + 2 and branch II, containing groups 3 + 4, indicating that in all these multi-locus phylogenies, the function of the individual selected proteins made little difference. However, in all three cases, but particularly in the 213 protein set [19], the functional diversity of the selected proteins was large.

Just like groups 1 and 2 in branch I, which are sister clades that evolved independently, groups 3 and 4 are sister clades in branch II, but with Pvio, one of six taxa in the "violaceum" complex [12] occupying a basal position to group 4. Group 4 taxa display major phenotypic innovations, such as two more cell types, cell-type proportioning, extensive slug migration and enhanced fruiting body robustness, which are not yet evident in the “violaceum” complex [18, 20]. To identify the molecular changes that caused these innovations, the genomes of taxa in the “violaceum” complex can now be considered as the baseline from which these changes occurred.

*Dcep*, one of four *D. polycephalum* isolates that make up the “polycephalum” complex is now the earliest diverging species in branch II. Although one might argue whether four isolates of the same species make up a “major division”, as suggested previously [12], we feel that the polycephalid isolates with their distinctive phenotype are likely to represent a group of cryptic species. Unlike all other non-group 4 species, polycephalids form very long actively migrating slugs, which unlike group 4 slugs do not show a pattern of prestalk and prespore cells [20]. Also unlike group 4 slugs, polycephalid slugs subdivide into many small fruiting structures with stalks that adhere tightly `along approximately their lower two-thirds. Similar to *P. violaceum* isolates, which are also grouped together as one species, due to their conspicuous purple branched fruiting bodies, the *D. polycephalum* isolates show considerable diversity in their SSU rDNA sequences [12, 24].

*Dcar*, member of the “polycarpum” complex [12], was previously located as sister to branch II in the SSU rDNA and α-tubulin phylogenies [10, 12]. The current 47 protein phylogeny confidently positions *Dcar* as outgroup to or earliest diverging species of group 2. With only two isolates of *Dcar* representing the “polycarpum” complex, its status as a “complex” is less convincing, particularly since it has no phenotypic features that set it apart from many species in groups 1–3. The Acytostelid *Aell*, previously positioned as outgroup to clade 2B with SSU rDNA, and as outgroup to group 2 with α-tubulin [10], is now firmly placed as outgroup to group 2. Group 2 consists of clade 2B, which consists of white Polysphondylids and other species with a cellular stalk, and clade 2A which contains only Acytostelids without cellular stalk. As before, this still presents a confusing history of stalk cell differentiation in Dictyostelia. *Dcar*, the outgroup to *Aell* and group 2 already have a cellular stalk, as do species in group 1 and branch II. This indicates that the cellular stalk evolved before the separation between branch I and II in taxa that are either extinct, or as yet undiscovered. The stalk was then lost twice, in *Aell* and in clade 2A, which likely involved changes in only one or a few genes. Interestingly, all Acytostelids, including *Aell*, have GC-rich genomes, but this is not the case for *Dcar* and *Ppal* (Fig. 2c). This suggests the possibility that an A/T to G/C non-synonymous mutation in a coding sequence, or an A/T to G/C conversion in a gene regulatory element may have caused the loss of the cellular stalk.The consensus phylogeny was also reproduced by four out of five subsets of nine or ten concatenated proteins from the 47-protein set, but only two proteins (rpa1 and smdA) out of the 47 set yielded the consensus tree on their own. The most common alternative topologies show *Dcar* as outgroup to group 1, or sister to the group 1 species *Ddem* (17 proteins), while the positions of *Pvio* and *Dlac* in branch II were reversed or joined into a single clade in nine proteins (Additional file 2, sheets 4 and 5). However, when these and other popular alternative topologies were individually constrained during inference of the 47 protein tree, their probability of the alternative topology being correct was at least 10,000 times lower than that of the consensus topology Table 1.

**Table 2:** Species and culture conditions

Species	Strain/isolate	Medium	Temperature
*Acytostelium ellipticum*	AE2	1/5^th^ SM + 0.5% charcoal	21 °C
*Acytostelium leptosomum*	FG12A	1/5^th^ SM +0.5% charcoal	25 °C
*Dictyostelium deminutivum*	MexM19A	1/3^rd^ LP + 0.5% charcoal	25 °C
*Dictyostelium polycarpum*	OhioWILDS	1/5^th^ SM	21 °C
*Dictyostelium polycephalum*	MY1-1	1/5^th^ SM	25 °C
*Polysphondylium violaceum*	P6	SM	21 °C

The species that were used for genome sequencing are listed with their preferred growth conditions. Full SM agar contains 10 g BactoTM peptone, 1 g yeast extract, 10 g glucose, 1 g MgSO_4_.7H_2_O, 2.2 g KH_2_PO_4_, 1.25 g Na_2_HPO_4_.2H_2_O and 15 g agar per liter, for 1/5^th^ SM all quantities, except the agar and phosphates are five-fold reduced. Full LP agar contains 1 g lactose, 1 g BactoTM peptone, 2.2 g KH_2_PO_4_, 1.25 g Na_2_HPO_4_.2H_2_O and 15 g agar per liter. For 1/3^rd^ LP the lactose and peptone quantities are three-fold reduced

The ability of specific proteins to reproduce the consensus phylogeny was only marginally correlated with the number of total or variable positions in the alignment, and did not appear to be correlated with protein function (involvement in a specific metabolic pathway). The reason why trees inferred from some proteins predict species relationships better than others is therefore unclear. The proteins that yielded trees with one erroneous node, mostly yielded consensus trees when combined with one or at most two proteins that yielded a different error. This implies that with a carefully selected set, 3–5 genes may be sufficient to generate robust species phylogenies. This allows the use of a gene amplification approach rather than whole genome sequencing as the means to correctly classify all known and newly to be discovered Dictyostelia. This is a low-cost alternative to whole genome sequencing, but also avoids problems with contamination of genome reads with bacterial DNA that rendered one of our six sequenced genomes completely and another partially useless. Since the genes used in this study are also present in representatives of the two major divisions Conosa and Lobosa of Amoebozoa (with *Acas* in Lobosa, and *PhyP* and Dictyostelia in Conosa) a similar approach could also assist to generate a robust phylogeny of Amoebozoa, to replace and/or complement the current very poorly resolved SSU rDNA phylogeny [25]. In future studies we will explore use of a multi-locus gene amplification approach for incorporation of most known Dictyostelia in the phylogeny.

## Conclusions

Accurate phylogenies are of elementary importance for understanding the directionality of organismal evolution. The lack of resolution and topology errors of single gene phylogenies progressively disappear when more genes are incorporated in the underlying alignments. However, such a multi-locus approach requires completely sequenced genomes, which, particularly for unicellular eukaryotes, are only sparsely available and poorly representative of the genetic breadth of eukaryotes.

The major division of Amoebozoa gave rise to at least two independent inventions of multicellularity, of which the Dictyostelia represent the best studied and phenotypically most diverse group. Previous phylogenies based on SSU rDNA and proteins from existing genomes only partially resolved relationships between major and minor clades. To resolve these relationships, we sequenced the genomes of six early diverging dictyostelid taxa and annotated 47 functionally diverse deeply conserved proteins. These sequences combined with orthologous sequences from six dictyostelid and three unicellular amoebozoan sequenced genomes were used both concatenated and individually for phylogenetic inferences. The extremely robust phylogeny derived from concatenated sequences highlights monophyly of Dictyostelia, but polyphyletic origins of its three genera. Somatic cells were present in the last common ancestor of Dictyostelia, but were likely lost twice independently in group 2. Only two proteins individually reproduced this consensus phylogeny, but sets of two or three proteins, which individually yielded minor errors, again reproduced the core phylogeny. This emphasizes the inherent error-prone nature of single gene phylogenies, but also indicates that reliable classification of taxa can be achieved by sequencing just a few genes. This will be particularly useful for broader and deeper sampling of taxa in Dictyostelia, Amoebozoan and other major eukaryote divisions, and for classification of newly discovered species.

## Methods

### Species, culture and genomic DNA preparation

All species were grown in association with *Escherichia coli* 281 using the culture media and culture temperatures listed in Table 2. After amoebas had cleared the bacteria, they were harvested in 10 mM phosphate buffer pH 6.5, washed three times and starved shaken at 21 °C and 150 rpm for 4 h to allow clearance of bacteria.

To prepare genomic DNA, cells were lysed in 62.5 mM EDTA, 1% (w/v) SDS and 1% (v/v) β-mercaptoethanol in 50 mM Tris pH 7.2, and incubated at 80 °C for 10 min. Genomic DNA was purified by extraction with phenol:chloroform:isoamylalcohol 25:24:1 pH 8.0 using phase-lock columns and ethanol precipitation. After resuspension in 0.1 mM EDTA in 1 mM Tris, pH 8.0, DNA was incubated for 3 h with 0.5 μg/μl RNAse A. Integrity of DNA was tested visually on a 1% TAE agarose gel.

### Genome sequencing and assembly

Six Illumina TruSeq DNA libraries were prepared from the six species listed in Table 2, using standard Illumina protocols followed by Pippin (Sage) size selection to a target insert size of 400 bp. The six libraries were barcoded and sequenced together on a single lane of an Illumina HiSeq 2500 platform using 150 base paired-end reads in rapid mode to yield approximately 120 M read pairs. Removal of adapter sequences and quality trimming of the reads was performed with fastq-mcf v1.1.2-537 and reads were assembled using the CLC-BIO assembler v4.2.0 (Qiagen) to yield the pre-filtering assemblies. The contigs were annotated using ncbi-blast + v2.2.28, which showed variable levels of contamination between genomes with *Escherichia coli* DNA. The reads were also aligned against the *E. coli* K12 genome, using bwa v0.7.5a [26], and a new assembly was prepared using all unaligned reads. The annotation of the contigs assembled from these *E.coli* filtered reads was again assessed using ncbi-blast + v2.2.28. The assembly statistics of the six genomes before and after filtering of *E.coli* sequences is listed in Additional file 1. Library preparation, sequencing and bioinformatic analyses of raw Illumina reads were carried out by Edinburgh Genomics at the University of Edinburgh (http://genomics.ed.ac.uk).

### Gene selection, identification of orthologs and gene model prediction

Forty *Dictyostelium discoideum* proteins larger than 200 amino acids were initially selected from enzymes mediating 14 biochemical pathways of primary metabolism, retrieved from the KEGG database [27], which were conserved between *D. discoideum*, *Polysphondylium pallidum* and *D. fasciculatum* [16]. This set was supplemented with an additional twelve conserved proteins with a range of cell biological functions. Putative orthologs were retrieved by BLASTp from the published genomes of *D. purpureum*, *D. lacteum*, *P. pallidum*, *Acytostelium subglobosum*, *D. fasciculatum, Physarum polycephalum* and *Entamoeba histolytica* and *Acanthamoeba castellani.*

The newly sequenced genome contigs of *D. polycarpum*, *D. polycephalum*, *D. deminutivum*, *A. ellipticum*, *A. leptosomum* and the genome contig sequences of *P. violaceum strain* QSvi11, which were available in Genbank, were archived in Artemis [28] and converted into a BLAST database. This database was queried for the presence of the coding genes for each of the 52 proteins using tBLASTn. The contig and coordinate information of the hit was used to retrieve a genomic DNA fragment from the Artemis archive with sufficient flanking sequence to allow reconstruction of the complete gene model. The gene models were manually predicted using DNAMAN software (Lynnon Corp., Quebec, Canada), guided by existing gene models for the published orthologous *Dictyostelium* genes. Further corrections were made when protein alignments suggested the presence of indels or inappropriately assigned start and stop codons (see Fig. 1).

### Phylogenetic inference

#### Protein sequence alignment and concatenation

All putative orthologs for each of the 52 proteins were aligned using M-coffee [29] version 10.00, using eight different alignment algorithms to generate a consensus multiple alignment. Sections with poor consensus alignment (<50%) and long insertions affecting few genes were deleted using Jalview v 2.8.2 [30]. Five proteins were rejected from further analysis due to insufficient consensus alignment. Inspection of the alignments and of trees inferred from individual alignments revealed that some species contributed paralogs or bacterial contaminants rather than orthologs to the alignment, which were replaced by orthologs, when available, or deleted. In alignments, paralogs stand out by being markedly divergent from genes in related species, while in trees paralogs end up in outgroup positions. The final alignments of the 47 proteins were concatenated using catfasta2phyml v1.0 (https://github.com/nylander/catfasta2phyml) yielding a total of 37,410 aligned amino acid positions. This alignment, shown in Additional file 6, was used in its entirety for phylogenetic inference, or divided into five subsets of nine or ten proteins.

#### Phylogenetic trees

Phylogenetic trees were inferred using MrBayes v3.2.2 [31] and RAxML version 8.1.20 [32] with either a mixed amino acid substitution model for the entire alignment, or with concatenated alignments partitioned into sections which contained genes with the same optimal amino acid substitution model. Each partition was then run under its optimal model. For all analysis, rate variation between sites was estimated by a gamma distribution with four rate categories and a proportion of invariable sites. Bayesian analyses were run for one to two million generations for alignments of individual genes and for 100,000 generations for concatenated alignments. The concatenated alignments already fully converged (SD of split frequencies = 0) at less than 10,000 generations. RaxML analyses were run with 100 bootstrap replicates. Trees were visualized using Figtree v.1.4.2 (http://tree.bio.ed.ac.uk/software/figtree/) and rooted at midpoint or on the outgroup of unicellular Amoebozoa.

#### Test of alternative topologies

Alternative tree topologies were investigated using the approximately unbiased (AU) test as implemented in the program CONSEL v0.20 [23]. Trees with constrained nodes, as shown in Additional file 5 were generated using RaxML, and the RaxML log likelihood values for the consensus tree topology and the alternative tree topologies were used as input for the AU test.

## Additional files

Additional file 1: Excel spreadsheet (.xlsx) listing assembly statistics of the six sequenced genomes before and filtering out of contamination with *E.coli* reads. (XLSX 16 kb)
Additional file 2: Excel spreadsheet (.xlsx) listing in: Sheet 1 “Protein data”: Identifiers or sequences of the 47 test proteins from published and newly sequenced genomes, respectively. Sheet 2 “GC_content”: GC-content of all test proteins. Sheet 3 “Gene_subsets”: Proteins contained in each of 5 tested protein subsets. Sheet 4 “Tree_properties”: Listing of aligned and variable positions of each of the 47 protein alignments and the number of non-consensual nodes and averaged posterior probabilities of the inferred tree. Sheet 5 “Alternative_topol.”: Listing of alternative non-consensual topological changes of each individual protein tree. Sheet 6 “protein function”: Test for correlation between protein function and tree errors. (XLSX 213 kb)
Additional file 3: Phylogenies inferred from 47 individual proteins. Consensus alignments of orthologous sequences of 47 proteins retrieved from 14 amoebozoan genomes were determined using M-coffee, with eight alignment algorithms. Regions with poor consensus alignment or with long insertions in only few proteins were deleted. Phylogenies were inferred by Bayesian inference using a mixed amino-acid substitution models with rate variation between sites estimated by a gamma distribution with a proportion of invariable sites. Analysis were run for one million generations. Trees were rooted at midpoint using Figtree (http://tree.bio.ed.ac.uk/software/figtree/), with posterior probabilities shown at the nodes. Panel A: proteins a-h; Panel B: proteins m-x. The consensus phylogeny of all 47 concatenated proteins (see also Fig. 2) is shown top left in Panel A. (PDF 156 kb)
Additional file 4: Tree error compensation by concatenation. Concatenated alignments of two or three proteins that individually yielded trees with a single non-consensual node at different positions were subjected to Bayesian inference as described for Additional file 3. Four out of five concatenated alignments (B-E) yielded the consensus tree (A). Only aco1 required two additional proteins to correct its topology errors. (PDF 122 kb)
Additional file 5: Alternative topologies. Full representation of the constrained alternative topologies to the consensus tree that were tested using the Approximately Unbiased test. See Table 1 and Fig. 5 of main text. (PDF 133 kb)
Additional file 6: Fasta file of the concatenated alignment of 47 proteins across 14 Amoebozoan taxa. (FAS 528 kb)
