# Supplementary material for: A core phylogeny of Dictyostelia inferred from genomes representative of the eight major and minor taxonomic divisions of the group
Source: BMC Evol Biol. 2016 Nov 17;16:251. doi: 10.1186/s12862-016-0825-7 (PMC5114724; doi:10.1186/s12862-016-0825-7)
Supplement: Additional file 3: — Phylogenies inferred from 47 individual proteins. Consensus alignments of orthologous sequences of 47 proteins retrieved from 14 amoebozoan genomes were determined using M-coffee, with eight alignment algorithms. Regions with poor consensus alignment or with long insertions in only few proteins were deleted. Phylogenies were inferred by Bayesian inference using a mixed amino-acid substitution models with rate variation between sites estimated by a gamma distribution with a proportion of invariable sites. Analysis were run for one million generations. Trees were rooted at midpoint using Figtree (http://tree.bio.ed.ac.uk/software/figtree/), with posterior probabilities shown at the nodes. Panel A: proteins a-h; Panel B: proteins m-x. The consensus phylogeny of all 47 concatenated proteins (see also Fig. 2) is shown top left in Panel A. (PDF 156 kb) [file 12862_2016_825_MOESM3_ESM.pdf]

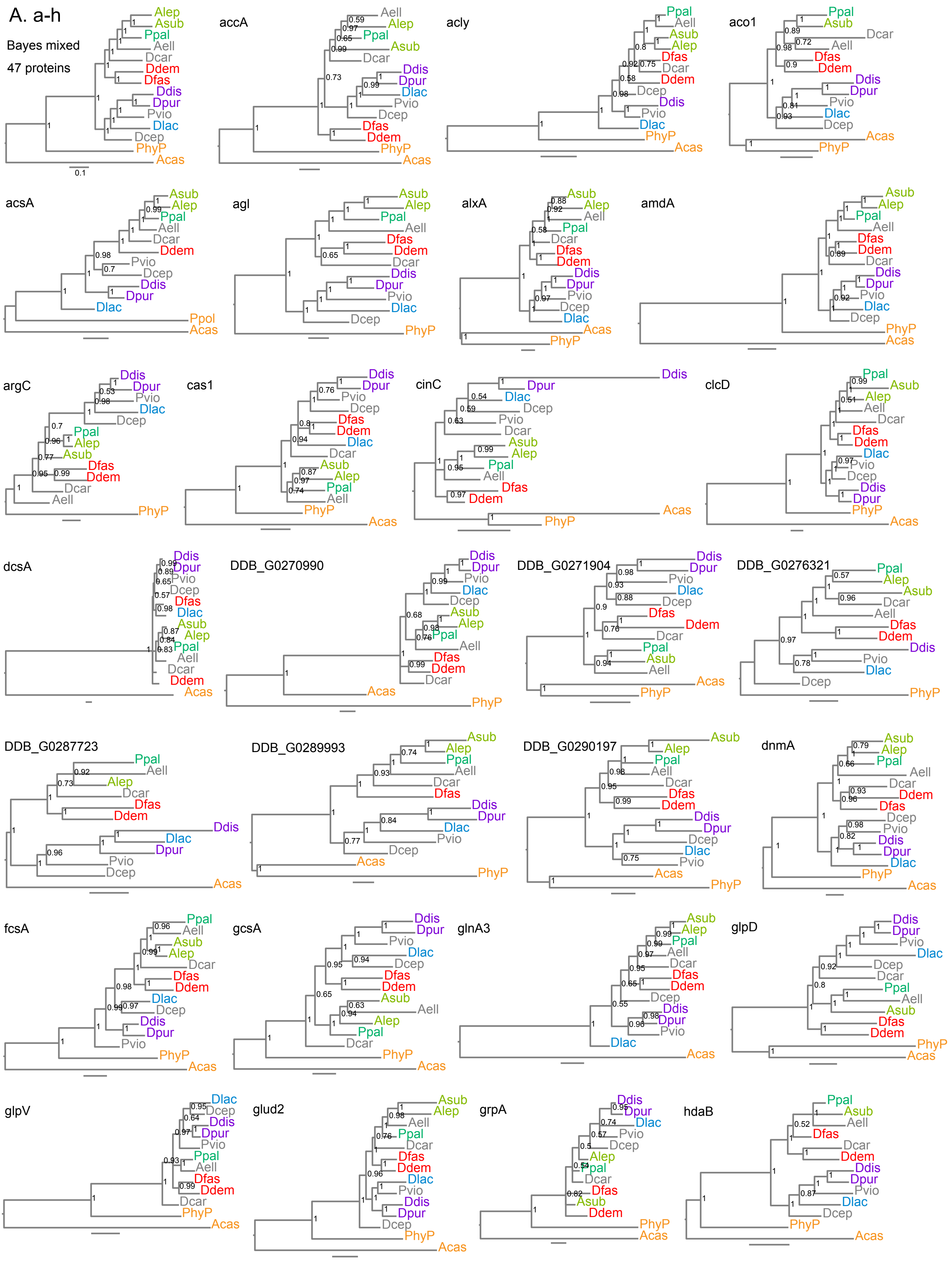
A. a-h
Bayes mixed
47 proteins
0.1
accA
acly
aco1
acsA
agl
alxA
amdA
argC
cas1
cinC
clcD
dcsA
DDB_G0270990
DDB_G0271904
DDB_G0276321
DDB_G0287723
DDB_G0289993
DDB_G0290197
dnmA
fcsA
gcsA
glnA3
glpD
glpV
glud2
grpA
hdaB

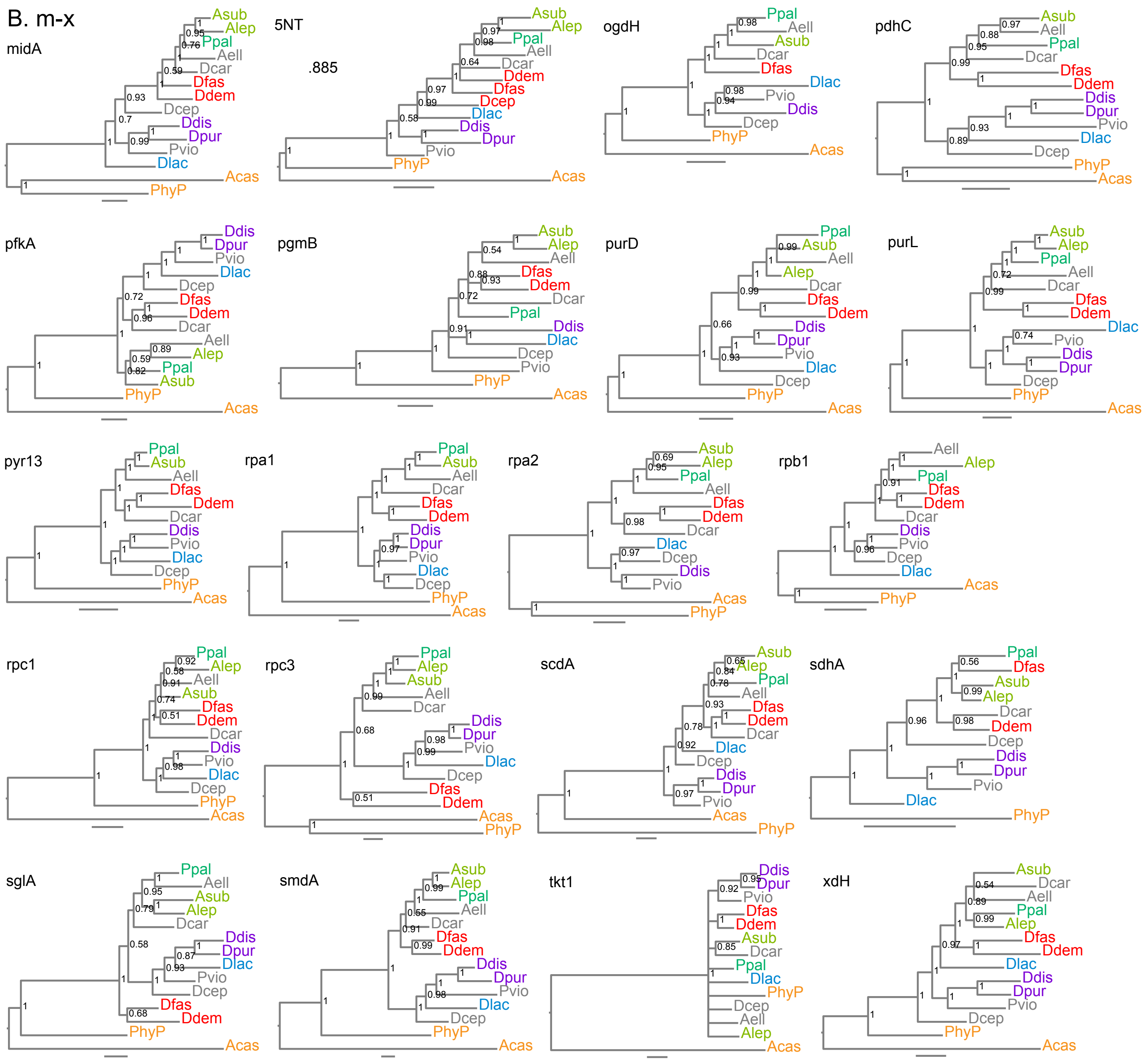

Additional file 3. Phylogenies inferred from 47 individual proteins

Consensus alignments of orthologous sequences of 47 proteins retrieved from 14 amoebozoan genomes were determined using M-coffee (Wallace et al., 2006), with 8 alignment algorithms. Regions with poor consensus alignment or with long insertions in only few proteins were deleted. Phylogenies were inferred by Bayesian inference (Ronquist and Huelsenbeck, 2003) using a mixed amino-acid substitution models with rate variation between sites estimated by a gamma distribution with a proportion of invariable sites. Analysis were run for 1 million generations. Trees were rooted at midpoint using Figtree (http://tree.bio.ed.ac.uk/software/figtree/), with posterior probabilities shown at the nodes. Panel A: proteins a-h; Panel B: proteins m-x. The consensus phylogeny of all 47 concatenated proteins (see also figure 2) is shown top left in Panel A.
