# Supplementary material for: A core phylogeny of Dictyostelia inferred from genomes representative of the eight major and minor taxonomic divisions of the group
Source: BMC Evol Biol. 2016 Nov 17;16:251. doi: 10.1186/s12862-016-0825-7 (PMC5114724; doi:10.1186/s12862-016-0825-7)
Supplement: Additional file 4: — Tree error compensation by concatenation. Concatenated alignments of two or three proteins that individually yielded trees with a single non-consensual node at different positions were subjected to Bayesian inference as described for Additional file 3. Four out of five concatenated alignments (B-E) yielded the consensus tree (A). Only aco1 required two additional proteins to correct its topology errors. (PDF 122 kb) [file 12862_2016_825_MOESM4_ESM.pdf]

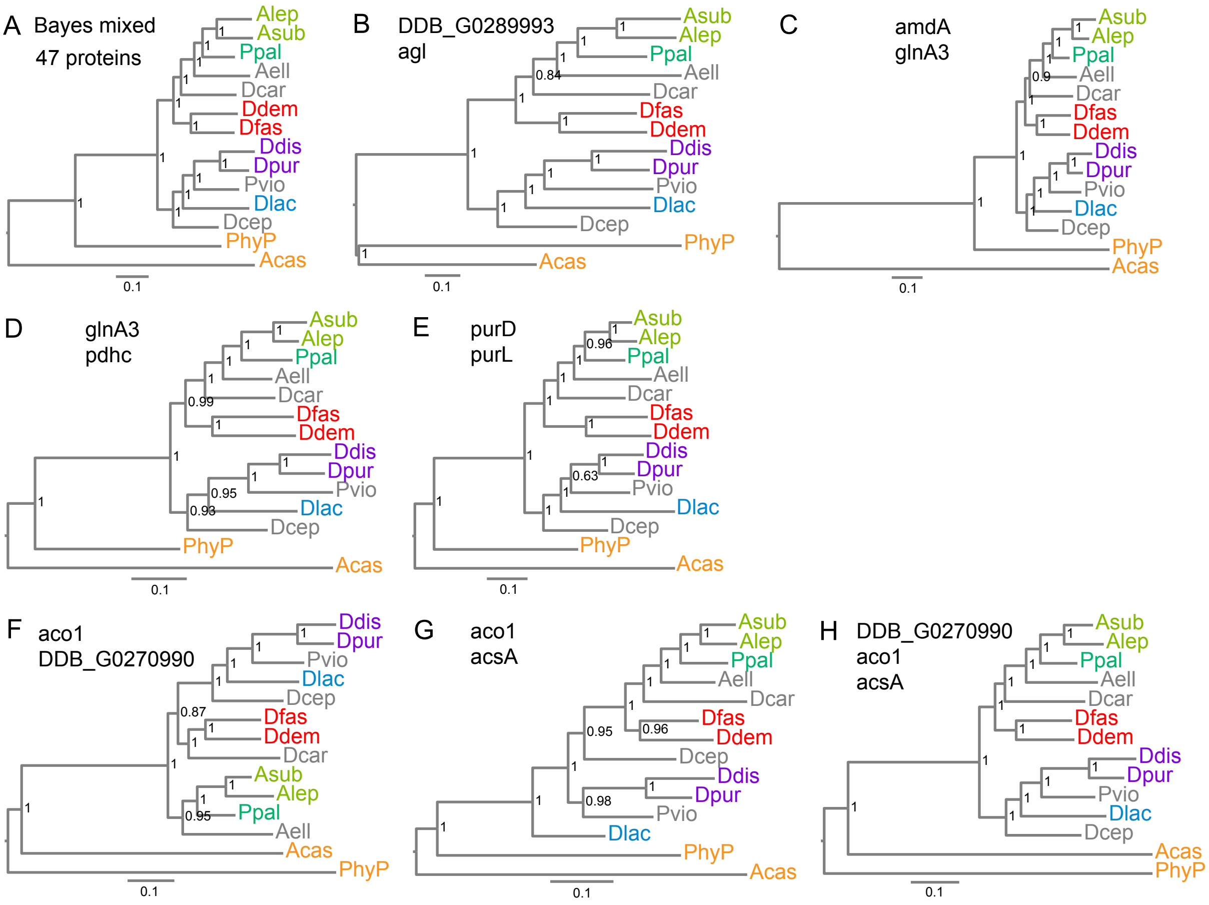

Additional file 4. Tree error compensation by concatenation

Concatenated alignments of two or three proteins, which individually yielded trees with a single non-consensual node at different positions, were subjected to Bayesian inference as described for Additional file 3. Four out of five concatenated alignments (B-E) yielded the consensus tree (A). Only aco1 required two additional proteins to correct its topology errors.
