# Supplementary material for: A core phylogeny of Dictyostelia inferred from genomes representative of the eight major and minor taxonomic divisions of the group
Source: BMC Evol Biol. 2016 Nov 17;16:251. doi: 10.1186/s12862-016-0825-7 (PMC5114724; doi:10.1186/s12862-016-0825-7)
Supplement: Additional file 5: — Alternative topologies. Full representation of the constrained alternative topologies to the consensus tree that were tested using the Approximately Unbiased test. See Table 1 and Fig. 5 of main text. (PDF 133 kb) [file 12862_2016_825_MOESM5_ESM.pdf]

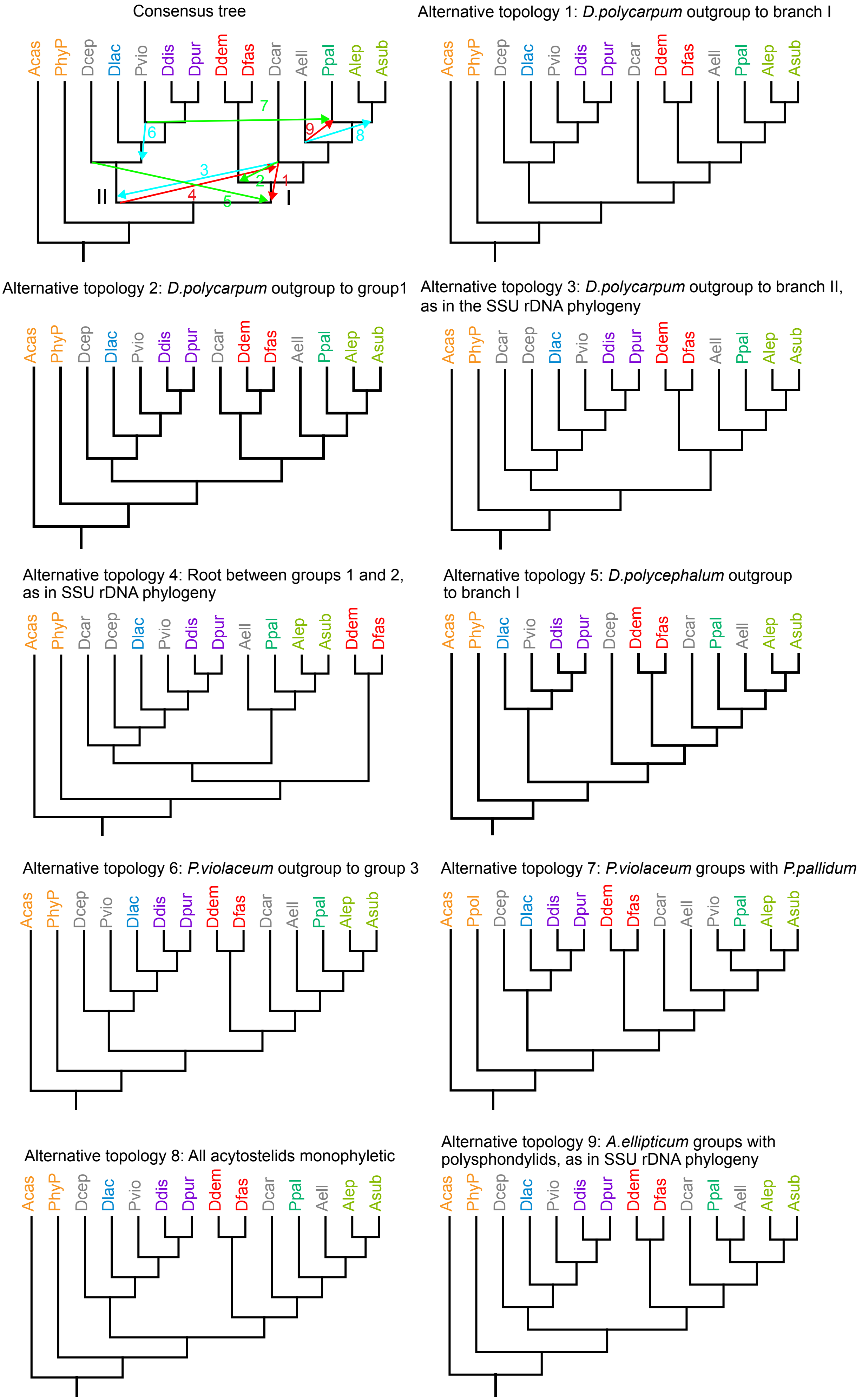

Additional file 5. Alternative topologies

Full representation of the constrained alternative topologies to the consensus tree that were tested using the Approximately Unbiased test. See table 2 and figure 5 of main text.
